# Pathophysiological mechanisms and therapeutic potential of E-selectin in pulmonary arterial hypertension

**DOI:** 10.3389/fimmu.2026.1754940

**Published:** 2026-07-10

**Authors:** Erha Lama, Yiyi Wang, Jiacheng Wu, Qian Ma, Hao Chen, Yalei Wang, Yulu Yang, Changhu Liu, Xiaofei Ni, Zihua Zhou

**Affiliations:** 1 Department of Cardiology, Union Hospital, Tongji Medical College, Huazhong University of Science and Technology, Wuhan, Hubei, China; 2 Hubei Key Laboratory of Biological Targeted Therapy, Union Hospital, Tongji Medical College, Huazhong University of Science and Technology, Wuhan, China; 3 Hubei Provincial Engineering Research Center of Immunological Diagnosis and Therapy for Cardiovascular Diseases, Union Hospital, Tongji Medical College, Huazhong University of Science and Technology, Wuhan, China; 4 Key Laboratory of Biological Targeted Therapy, Huazhong University of Science and Technology, Ministry of Education, Wuhan, Hubei, China; 5 Department of Cardiology, Renmin Hospital of Wuhan University, Wuhan, China

**Keywords:** endothelial cell dysfunction, E-selectin, inflammatory response, pulmonary arterial hypertension, therapeutic targets, vascular remodeling

## Abstract

Pulmonary arterial hypertension (PAH) is a life-threatening pulmonary vascular disease characterized by progressive obstructive remodeling of the small pulmonary arteries. Its pathophysiological core involves perivascular inflammation, endothelial dysfunction, abnormal vascular wall proliferation and *in situ* thrombosis, arising from interactions between genetic susceptibility and environmental factors. Recent studies have demonstrated the central role of chronic inflammation in the development and progression of PAH. E-selectin, an endothelial-specific adhesion molecule, plays a critical role in mediating the initial adhesion and migration of leukocytes and in triggering the inflammatory cascade. Clinical and experimental studies have demonstrated that E-selectin levels are significantly elevated in both the circulation and local lung tissues of patients with PAH, and that these levels are positively correlated with disease severity. Although observational data have not yet directly established a causal relationship, the combination of its well-recognized proinflammatory properties suggests that E-selectin may function not only as an inflammatory marker but also as a potential key mediator driving pulmonary vascular inflammation and remodeling. This review systematically discusses the biological characteristics of E-selectin and its expression regulation in PAH, further explores the molecular and cellular mechanisms by which it contributes to vascular remodeling, and focuses on potential therapeutic strategies targeting E-selectin, with the aim of providing a new perspective for precision anti-inflammatory treatment in PAH.

## 1 Introduction

Pulmonary arterial hypertension (PAH) is a heterogeneous group of clinical diseases characterized by abnormally high pulmonary artery pressures, which ultimately results in exercise intolerance, progressive dyspnea, exertional syncope, right heart failure, and even death (1). The pathogenesis of PAH is complex and involves a combination of genetic, epigenetic, and environmental factors (hypoxia, inflammation, oxidative stress, and drug exposure). Epidemiologic data indicate that the prevalence of PAH ranges from an estimated 1% in the general population to as high as 10% in people over the age of 65 years (2). The five-year mortality rate for patients with PAH is as high as 50% (1). This high mortality has earned PAH the moniker “the cancer of cardiovascular diseases.” According to the 2022 ESC/ERS guideline update, the diagnostic criteria for PAH include the following: a mean pulmonary arterial pressure of > 20 mmHg at rest (updated from > 25 mmHg) and a pulmonary vascular resistance (PVR) of > 2.0 Wood units (updated from > 3.0 Wood units) (3, 4). Pulmonary hypertension (PH) a broader term encompassing all five categories, is currently classified into five categories based on etiology and hemodynamic characteristics: (1) Arterial PH; (2) left heart disease-associated PH; (3) pulmonary disease and hypoxia-correlated PH; (4) chronic thromboembolic PH; (5) PH due to unexplained and/or multifactorial mechanisms (5, 6). Historically, the natural history of PAH was characterized by a dismal prognosis. The advent of targeted therapies—including endothelin receptor antagonists, phosphodiesterase type 5 inhibitors, prostacyclin analogs, and soluble guanylate cyclase stimulators—has significantly improved patient survival (7). However, these drugs primarily alleviate functional obstruction caused by pulmonary vasoconstriction and have limited efficacy in reversing pulmonary vascular remodeling, thereby failing to fundamentally halt the progression of PAH (1). Given the limitations of current vasodilatory therapies, PAH research is pivoting toward novel targets that address the disease’s underlying pathophysiological drivers, including metabolic reprogramming, epigenetic modulation, and growth factor signaling—most notably exemplified by sotatercept (3, 8).

Recent studies suggest that perivascular inflammatory infiltrates of mixed inflammatory cells often precede the structural pulmonary vascular remodeling, supporting the notion that maladaptation of the inflammatory and immune systems exists and contributes to remodeling (9). In particular, perivascular inflammatory responses significantly contribute to vascular remodeling in PAH (10, 11). Extensive infiltration of inflammatory cells has been observed in the perivascular regions of pulmonary arteries in both clinical PAH patients and animal models (12). These infiltrating cells release copious cytokines and inflammatory mediators, which not only promote endothelial cell apoptosis but also the proliferation and migration of smooth muscle cells and fibroblasts (13). In addition, inflammation and thrombosis are closely linked in a bidirectional, mutually reinforcing manner. First, activated endothelial cells upregulate adhesion molecules, which facilitate the recruitment of leukocytes, including neutrophils and monocytes, to the site of vascular injury. Once recruited, these inflammatory cells release tissue factor, procoagulant microparticles, and pro-inflammatory cytokines, thereby promoting a prothrombotic state (14, 15). Second, recruited monocytes can differentiate into macrophages that produce matrix metalloproteinases and reactive oxygen species, further damaging the endothelium and exposing subendothelial tissue factor, which triggers the coagulation cascade (16). Third, neutrophils can release neutrophil extracellular traps, which provide a scaffold for platelet adhesion and fibrin deposition, directly promoting thrombus formation. In Chronic thromboembolic pulmonary hypertension (CTEPH), persistent thromboembolic obstruction induces endothelial injury and inflammatory responses, which in turn drive thrombosis by enhancing leukocyte recruitment, promoting platelet activation, and upregulating coagulation responses. Conversely, cellular and molecular events during thrombosis further exacerbate pulmonary vascular endothelial injury, inflammation, and oxidative stress, thereby leading to vascular remodeling, increased vascular resistance, and elevated pulmonary arterial pressure (15, 17). By targeting the initial inflammatory cascade, specifically the adhesion molecules that mediate leukocyte-endothelial crosstalk, it may be possible to arrest both vascular remodeling and pro-thrombotic progression. Among the numerous inflammatory mediators, E-selectin, a key marker of endothelial activation and an initial tethering molecule for leukocyte transendothelial migration, may constitute a critical link between inflammation and pulmonary vascular remodeling in PAH (18, 19). Under hypoxic conditions or in response to inflammatory factors, activated endothelial cells upregulate E-selectin expression through the nuclear factor-κB (NF-κB) signaling pathway. E-selectin then binds to sialyl Lewis X antigens on the surface of neutrophils, facilitating their transmigration across endothelial junctions toward sites of injury, thereby inducing perivascular inflammation and promoting thrombosis through fibrin deposition (20, 21). E-selectin-mediated neutrophil-endothelial interactions involve activation of the S100A9-RAGE-PI3K-AKT pathway, which contributes to endothelial dysfunction (18, 19). The migration of neutrophils to sites of inflammation is a highly ordered, multistep process, involving (1) adhesion and rolling, (2) activation, (3) firm adhesion, and (4) transendothelial migration (20). In a normal immune response, this mechanism serves to contain and control infections. However, excessive E-selectin-induced recruitment of inflammatory cells may cause extensive tissue damage, leading to various pathological conditions, such as lung disease, thromboembolic disease, cardiovascular disease, and tumor metastasis (10, 22). In the context, E-selectin emerges as a compelling, yet under-explored, therapeutic target that uniquely bridges the inflammatory and structural dimensions of PAH pathogenesis.

## 2 Methods

### 2.1 Literature search strategy

A systematic literature search was conducted from June 2025 to April 2026 across PubMed/MEDLINE, Web of Science, Scopus, and Cochrane Library databases. The search covered publications from January 1989 to April 2026. The following MeSH terms and keywords were used in various combinations: “E-selectin,” “SELE,” “CD62E,” “ELAM-1,” “endothelial leukocyte adhesion molecule-1,” “pulmonary arterial hypertension,” “pulmonary hypertension,” “PAH,” “chronic thromboembolic pulmonary hypertension,” “CTEPH,” “thrombosis,” “inflammation,” “neutrophil,” “endothelial dysfunction,” “NF-κB,” “integrin,” “targeted therapy”, and “immunotherapy.” Additional articles were identified through manual screening of reference lists of included studies and relevant review articles.

### 2.2 Inclusion and exclusion criteria

Studies were included if they were: (1) original research articles, systematic reviews, meta-analyses, or clinical trials; (2) published in English; (3) focused on E-selectin biology, its role in inflammation and leukocyte adhesion, and/or its involvement in pulmonary hypertension pathogenesis. Studies were excluded if they were conference abstracts without full-text data, non-English publications, or not directly relevant to E-selectin or pulmonary hypertension.

### 2.3 Study selection and data extraction

Two independent reviewers (E.L. and C.L.) screened titles and abstracts, followed by full-text assessment. Discrepancies were resolved through discussion or consultation with a third reviewer (Y.W.-A. and Z.Z.). Data extracted included: first author, year of publication, study type, experimental models (cell types, animal models, clinical samples), key findings related to E-selectin expression and signaling, and relevance to pulmonary vascular remodeling, inflammation, and thrombosis.

A total of 98 articles were ultimately included in this review, covering basic biology of E-selectin, signal transduction pathways, pulmonary arterial hypertension pathophysiology, inflammatory mechanisms, and therapeutic targeting strategies.

## 3 Basic biological properties of E-selectin

Selectins are calcium-dependent type I transmembrane glycoproteins that recognize and bind to specific sugar groups on the surface of immune cells (22). Based on the order of their discovery, selectins are primarily categorized into LECAM-1 (L-selectin), LECAM-2 (E-selectin), and LECAM-3 (P-selectin) (22). Within the selectin family, P-selectin is primarily expressed on vascular endothelial cells and platelets, E-selectin is mainly expressed on vascular endothelial cells, and L-selectin is predominantly expressed on leukocytes (22). P-selectin and E-selectin expressed on vascular endothelial cells play distinct roles in acute and chronic inflammation. P-selectin is stored in Weibel-Palade bodies of endothelial cells and α-granules of platelets, and is rapidly translocated to the cell surface within minutes in response to inflammatory stimulation, making it the earliest selectin involved in leukocyte rolling. It binds with high affinity to P-selectin glycoprotein ligand-1 (PSGL-1), thereby initiating rapid rolling, and thus predominantly mediates early leukocyte rolling during acute inflammation (23). By contrast, E-selectin is not pre-stored in intracellular vesicles; its expression depends on gene transcription and *de novo* protein synthesis. It is first expressed 4–6 hours after inflammatory cytokine stimulation and reaches peak expression within 24 hours, representing a delayed yet sustained response (24). E-selectin mediates slower and more stable rolling, facilitates subsequent integrin activation and firm adhesion, and is therefore more critical for chronic or persistent inflammation.

E-selectin (LECAM-2, CD62E) was first discovered in the 1980s. Its primary function is to mediate leukocyte adhesion and stabilization on endothelial cells during inflammatory responses. Under resting conditions, endothelial cells express little to no E-selectin, maintaining the endothelium in an inactive state (25). However, upon stimulation by pro-inflammatory factors (tumor necrosis factor-α [TNF-α], lipopolysaccharide [LPS], interleukin-1β [IL-1β], E-selectin expression is markedly upregulated (26). This induction facilitates adhesion of neutrophils and their migration underneath the vascular endothelium, resulting in a perivascular inflammatory response (13). E-selectin exists in two forms: membrane-bound and soluble. Membrane-bound E-selectin is expressed on the surface of activated endothelial cells and mediates leukocyte rolling and adhesion. In contrast, soluble E-selectin in the circulation is primarily generated through proteolytic shedding or alternative splicing and is considered a biomarker of endothelial activation and inflammatory status (27). Although soluble E-selectin may modulate leukocyte-endothelial interactions, its biological functions remain less well characterized than those of membrane-bound E-selectin.

### 3.1 Molecular structure and functional domains

**Figure 1:**
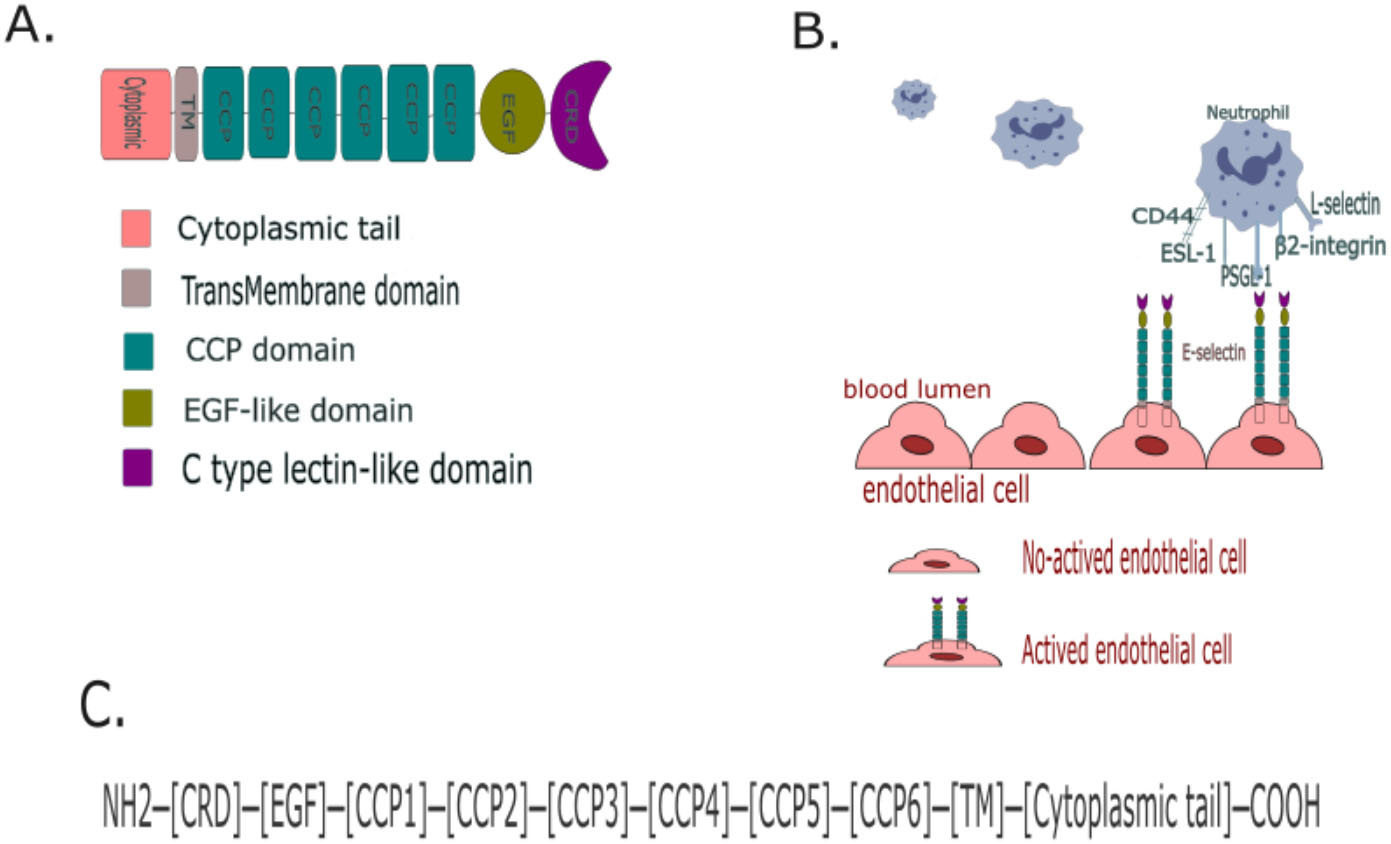
The structure and ligand types of E-selectin. **(A, C)** The domains of E-selectin. **(B)** Activated endothelial cells express E-selectin, while its ligands are expressed on neutrophils. CRD, C-type lectin-like domain; EGF, epidermal growth factor; CCP, complement control protein; TM, Transmembrane domain; PSGL-1, P-selectin glycoprotein ligand-1; ESL-1, E-selectin glycoprotein ligand-1.

E-selectin consists of five primary domains. These include an N-terminal C-type lectin-like domain(CRD), an epidermal growth factor (EGF)-like domain, six complement control protein (CCP) repetitive sequence domains, a transmembrane domain, and a short cytoplasmic tail region (**Figure 1**) (22, 28).

Among these, the CRD domain is the crucial region for E-selectin ligand binding and is located at the outermost N-terminal portion of the protein. This domain specifically recognizes and binds to various glycoprotein ligands on the surface of leukocytes, including P-selectin glycoprotein ligand-1 (PSGL-1), E-selectin glycoprotein ligand-1 (29), L-selectin, CD44, CD43, β2-integrins. The CRD has a Ca^2+^-binding site, which is crucial for maintaining the correct conformation required for ligand binding (30). The EGF-like domain, located between the C-type lectin structural domain and the CCP repeat sequence domain, contributes to molecular folding and stabilization. It may also regulate the affinity of E-selectin for ligand binding, although its precise role remains unclear (31). The CCP repeats typically contains multiple short, highly conserved complement control protein repeats that contribute to the correct conformation and proper localization of E-selectin at the cell membrane and may also play a role in signal transduction. The transmembrane domain connects the extracellular domain of E-selectin to the cytoplasmic tail region, anchoring the entire molecule.

### 3.2 Endothelial cell activation and E-selectin expression

Endothelial cells form a highly differentiated monolayer lining the inner surface of blood vessels, directly exposed to blood-borne mediators, biomechanical stimuli, and circulating blood cells. Disruption of endothelial cell structure and function is a crucial mechanism underlying the development of cardiovascular disease (32). Studies have demonstrated that endothelial cells are not merely an intravascular lining and a semipermeable barrier separating blood from tissue (33). A hallmark of endothelial cell activation is the increased expression of E-selectin in response to hypoxia and inflammatory stimuli (34). Hypoxia-induced endothelial activation, in particular, is a direct process. As described by Michiels et al., the underlying molecular mechanism involves a hypoxia-induced decrease in mitochondrial respiratory chain activity and subsequent ROS generation, which activates downstream pathways and ultimately upregulates adhesion molecule expression (35). E-selectin expression is markedly upregulated 2–4 h following endothelial cell activation. Its N-terminal CRD recognizes and binds to sLex on neutrophils and lymphocytes, promoting endothelial cell-neutrophil adhesion, and transendothelial migration (20). Activation of the NF-κB signaling pathway plays a crucial role in regulating E-selectin expression (36). Studies have indicated that the E-selectin promoter region contains NF-κB binding sites. Upon endothelial cell activation, NF-κB subunits particularly the p50/p65 heterodimer are activated and translocated into the nucleus (37). Immunoprecipitation and UV cross-linking experiments further confirmed that the p50/p65 heterodimer could directly bind to the NF-κB site of the E-selectin promoter (38), thereby activating transcription of the E-selectin gene and upregulating its expression. Currently, E-selectin-mediated rolling signaling to neutrophils induces their secretion of myeloid-related proteins 8/14, which results in the activation of high-affinity β2-integrins and binding to endothelial intercellular adhesion molecule-1 (ICAM-1) (39, 40). This results in firm adhesion of neutrophils to endothelial cells and subsequent transendothelial migration, thereby propagating the inflammatory response.

Notably, the upstream stimuli driving E-selectin expression are disease-specific: in acute infections such as COVID-19 (41). The expression of E-selectin may be triggered by newly generated autoantibodies (e.g., antiphospholipid antibodies). In chronic inflammatory diseases such as rheumatoid arthritis (42), it is driven by persistent pro-inflammatory cytokines (e.g., TNF-α, IL-6) and oxidative stress. Although the initiating signals differ, they ultimately converge on conserved signaling pathways such as NF-κB to activate E-selectin transcription. Therefore, E-selectin is not merely an effector molecule of endothelial dysfunction; its expression level also integrates and reflects the net effect of multiple upstream pathological stimuli, making it a critical hub that links specific etiologies, shared molecular mechanisms, and final vascular pathological phenotypes. Furthermore, according to recent reviews and experimental studies, pulmonary vascular endothelial dysfunction in patients with PAH is characterized by impaired vasodilation and anticoagulant properties, increased oxidative stress, upregulation of adhesion molecules, including E-selectin, ICAM-1, and vascular cell adhesion molecule 1 (VCAM-1), as well as abundant release of cytokines and chemokines. These alterations promote leukocyte recruitment, exacerbate endothelial-immune cell interactions, and impair vascular repair function, thereby contributing to *in situ* thrombosis and pulmonary vascular remodeling. Therefore, in the context of PAH, elevated E-selectin levels reflect a systemic state of inflammation and endothelial dysfunction rather than an isolated molecular alteration (9).

### 3.3 E-selectin-mediated signaling pathways

Studies indicate that E-selectin can bind to multiple leukocyte ligands, including PSGL-1, CD43, CD44, ESL-1, β2 integrin, and L-selectin. Among these, the most critical E-selectin ligand on neutrophils is PSGL-1 (29, 30). E-selectin binding to PSGL-1 not only mediates cell adhesion and rolling but also activates multiple downstream signaling pathways, regulating neutrophil activation, adhesion, and migration. Specifically, upon E-selectin-PSGL-1 binding, neutrophil Src family kinases are rapidly activated, mediating Syk activation and initiating three key downstream signaling pathways.

#### 3.3.1 PLCγ2-p38 mitogen-activated protein kinase signaling pathway

**Figure 2:**
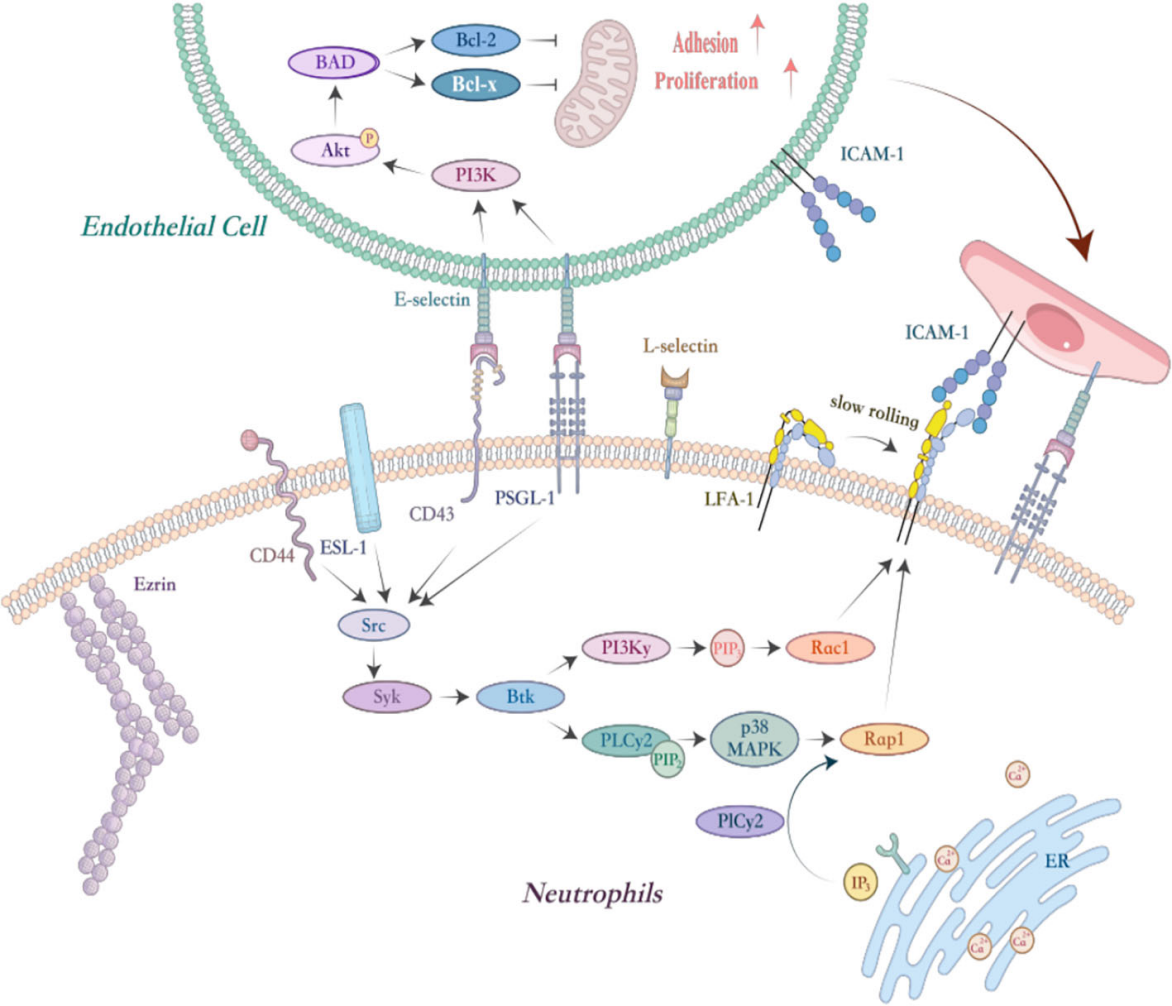
E-selectin binds to ligands on the surface of neutrophils and activates the downstream signaling pathways, which in turn activate Rac1 and Rap1. This process dynamically reorganizes the cytoskeleton, shifting the LFA-1 portion of β2-integrin from a low to a high affinity state, enabling its binding to endothelial intercellular adhesion molecule-1 (ICAM-1). This series of reactions allows neutrophils to adhere more firmly to endothelial cells and migrate across the endothelium, thereby triggering an inflammatory response. PI3K, phosphatidylinositol 3-kinase; Akt, protein kinase B; BAD, Bcl-2-associated death promoter, inhibits mitochondrial apoptosis; Bcl-2/Bcl-x, anti-apoptosis-associated protein; Src, tyrosine kinase; Syk, splenic tyrosine kinase; Btk, Bruton’s tyrosine kinase; PLCγ2, phospholipase Cγ2; p38 MAPK, p38 mitogen-activated protein kinase; PIP2/PIP3, phosphatidylinositol diphosphate/phosphatidylinositol triphosphate; IP3, inositol triphosphate; Ezrin, an actin-binding protein.

Following Syk activation, Bruton’s tyrosine kinase (Btk) from the Tec family is further activated, along with phospholipase Cγ2 (PLCγ2) (43, 44). PLCγ2 mediates the release of calcium ions, thereby activating the p38 MAPK pathway (45, 46). Notably, PLCγ2 is essential for the complete phosphorylation of p38 MAPK (43). Activation of the p38 MAPK signaling pathway induces the activation of β2-integrin (LFA-1) on neutrophils, thereby promoting slow rolling of neutrophils (**Figure 2**) (47).

#### 3.3.2 PI3K pathway

Binding of L-selectin and PSGL-1 to E-selectin induces PI3K activation. Activated PI3K catalyzes the phosphorylation of phosphatidylinositol-4,5-bisphosphate (PIP2) on the cell membrane (48), generating phosphatidylinositol-3,4,5-trisphosphate (PIP3). Moreover, PIP3 mediates the downstream cytoskeletal regulator Vav, thereby activating the GDP-GTP exchange activity of Rac1 protein (a Rho family GTPase). This results in neutrophil cytoskeletal reorganization, which, in turn, promotes cell-cell adhesion (49). Additionally, PI3K can activate Akt (protein kinase B), which phosphorylates Ser136 of the driver of death (BAD), thereby releasing the apoptosis inhibitory proteins Bcl-2 and Bcl-x. By inhibiting mitochondrial apoptosis, this pathway suppresses endothelial cell apoptosis, contributing to vascular remodeling (**Figure 2**) (50, 51).

#### 3.3.3 S100A9-RAGE-PI3K-AKT pathway

**Figure 3:**
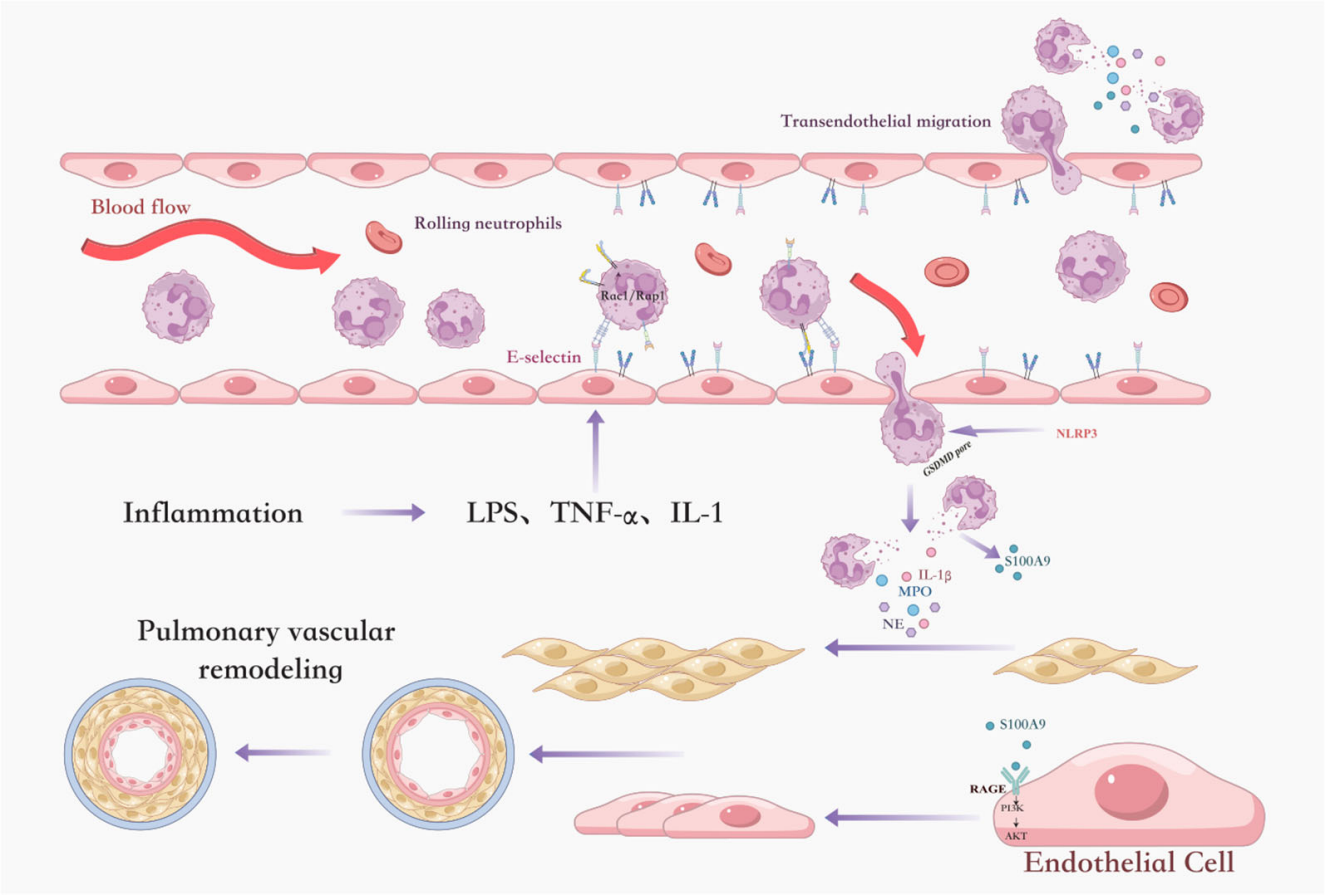
The cascade of neutrophil recruitment involving rolling on endothelial cells, adhesion, transendothelial migration into the subendothelial compartment, ultimately resulting in perivascular inflammation. LPS, lipopolysaccharide; TNF-α, tumor necrosis factor-alpha; IL-1, interleukin-1; NLRP3, NLR family pyrin domain containing 3; S100A9, S100 calcium-binding protein A9; MPO, myeloperoxidase; NE, neutrophil elastase; IL-1β, Interleukin-1 beta; GSDMD, gasdermin D; RAGE, receptor for advanced glycation end-products; PI3K, phosphoinositide 3-kinase; AKT, AKT serine/threonine kinase.

E-selectin on endothelial cell surfaces binds to neutrophils, activating the NLRP3 inflammasome and inducing caspase-1 cleavage, which triggers transient GSDMD pore formation and subsequent release of S100A9. This pro-inflammatory protein contributes to endothelial dysfunction and promotes PAH progression (18, 19). Additionally, Rong et al. demonstrated that caspase-8 promotes pulmonary vascular remodeling via the NLRP3/caspase-8/IL-1β axis in macrophages, and that caspase-8 inhibition attenuates PAH (52). Fais and Lahm further showed that the NLRP3 inflammasome also contributes to myocardial dysfunction and right heart failure through activation of M1-type CCR^2+^ macrophages in the right ventricle (53). Consequently, the E-selectin/E-selectin ligand axis represents a promising anti-inflammatory therapeutic target for reversing vascular remodeling (**Figure 3**).

## 4 Research evidence linking E-selectin to PAH

### 4.1 Changes in E-selectin levels in PAH

Pulmonary vascular remodeling is a crucial pathological feature of PAH and is closely correlated with abnormal death of pulmonary vascular cells and perivascular inflammatory responses. Studies indicate that abnormal death of pulmonary vascular cells can activate immune cell adhesion and release of inflammatory mediators, thereby further exacerbating the remodeling process (54). A study investigating the effects of ruxolitinib on pulmonary vascular remodeling found that it increased cyclic guanosine monophosphate levels in human umbilical vein endothelial cells, and decreased TNF-α-induced E-selectin expression.

Consequently, pulmonary vascular remodeling may be correlated with E-selectin expression (55). In patients with PAH, serum ELISA detected significantly elevated levels of soluble E-selectin. Concurrently, circulating endothelial cell counts in serum were markedly increased. These findings suggest endothelial cell activation, proliferation, and vascular remodeling may occur in PAH, rather than solely endothelial cell shedding solely endothelial cell shedding (56). In lung tissue specimens from patients with idiopathic PAH, the expression levels of E-selectin, ICAM-1, and VCAM-1 were markedly upregulated in pulmonary arterial endothelial cells (57). Furthermore, in hypoxia-induced PAH animal models, immunohistochemical and Western blot analyses demonstrated significantly elevated endothelial expression of E-selectin (58). Collectively, these results suggest that E-selectin may contribute to pulmonary vascular remodeling by mediating inflammatory responses and endothelial cell activation.

### 4.2 Current status of clinical research on E-selectin gene polymorphisms

Although current studies have not demonstrated a direct correlation between E-selectin gene polymorphisms and PAH, genetic variants in cell adhesion molecules, including E-selectin, have been significantly associated with cardiovascular diseases. Three single-nucleotide polymorphisms within the E-selectin gene have been significantly associated with cardiovascular diseases or their risk factors (59). These include an A-to-C transversion in exon 4, resulting in a serine-to-arginine substitution at codon 128, S128R (60); a G-to-T polymorphism in the untranslated region of exon 2, G98T (61); and a C-to-T transversion in exon 11, resulting in a leucine-to-phenylalanine substitution at codon 554, L554F (62–64). In hypertensive patients, the homeostatic regulation between nitric oxide and E-selectin is disrupted (64); a similar mechanism may also contribute to PAH pathogenesis. Furthermore, in a clinical study of coronary atherosclerosis, Yoshida et al. demonstrated that the E-selectin S128R polymorphism alters leukocyte-endothelial interactions and the biochemical and functional consequences of these interactions, which may contribute to the pathogenesis of myocardial infarction (65). These findings suggest that E-selectin genetic variants may indirectly influence susceptibility to or severity of PAH, warranting further mechanistic and association studies in PAH populations.

## 5 Mechanisms of E-selectin in PAH pathophysiology

### 5.1 E-selectin and activation of inflammatory signals

Pulmonary vascular remodeling, a hallmark of PAH in both patients and animal models, is often preceded and potentially driven by perivascular inflammatory infiltration (66). E-selectin, a key molecule in the early stages of inflammation, mediates the initial adhesion of leukocytes to the endothelium (rolling) and their subsequent crossing of the vascular wall during inflammation, and is a crucial initiating molecule for vascular endothelial cell-neutrophil interaction (67). E-selectin expression is restricted to activated endothelial cells. Induced by inflammatory mediators (TNFα, IL-1β, and lipopolysaccharide), activated endothelial cells upregulate E-selectin expression through the NF-κB signaling pathway (38). The binding of E-selectin to sLex on the surface of neutrophils prompts slow migration of neutrophils into the subendothelium, which in turn triggers perivascular inflammation. Moreover, the inflammatory response induces the expression of S100A9, an inflammation-associated protein. This protein amplifies the inflammatory cascade and vascular injury, ultimately leading to endothelial dysfunction and vascular remodeling (19).

### 5.2 E-selectin and vascular remodeling

Pulmonary vascular remodeling has been consistently recognized as a central pathological feature and a key pathological change in PAH (68). It is primarily characterized by perivascular inflammatory cell infiltration and dysfunction of endothelial and smooth muscle cells in the pulmonary vasculature (9). First, perivascular inflammation drives pulmonary vascular remodeling by affecting pulmonary vascular hemodynamics and altering the phenotype of pulmonary vascular cells, which in turn exacerbates pulmonary vascular resistance (13). In lung tissues from patients with PAH, the expression levels of E-selectin and ICAM-1 were significantly upregulated in distal pulmonary artery endothelial cells (57). Second, resistance to endothelial apoptosis is a fundamental phenotypic shift that occurs in pulmonary vascular cells in PAH (69). In a hypoxia-induced mouse model of PAH, Song et al. found that inhibiting mitochondrial depolarization through the E-selectin pathway induced the expression of the anti-apoptotic protein Bcl-2, which inhibited apoptosis of pulmonary artery endothelial cells, resulting in proliferation of the pulmonary artery intima (70). Both excessive apoptosis (in early PAH) and resistance to apoptosis (during disease progression) of pulmonary artery endothelial cells promote pulmonary vascular remodeling (71). Consequently, E-selectin, as a critical endothelial–neutrophil adhesion molecule, may drive pathologic vascular remodeling by mediating perivascular inflammatory responses.

#### 5.2.1 E-selectin and endothelial cells

One of the pathological hallmarks of PAH is pulmonary artery endothelial cell dysfunction, which disrupts vascular homeostasis by adopting a pro-inflammatory phenotype transformation (57). E-selectin, a key marker of endothelial cell activation (72), plays a crucial role in initiating inflammation in this context. In PAH development, multiple injurious factors lead to the conversion of pulmonary artery endothelial cells from a resting state to an activated state. The state is characterized by a decrease in NO and prostacyclin synthesis, increased secretion of endothelin-1, and aberrant release of various inflammatory factors, which ultimately triggers persistent vasoconstriction, abnormal proliferation of medial smooth muscle cells, and significant pulmonary vascular remodeling (73). E-selectin, a cell adhesion molecule, serves as a hallmark of endothelial cell-specific activation. Under resting conditions, endothelial cells barely express E-selectin; however, in response to stimulation by PAH-associated inflammatory factors (TNF-α, IL-1β) or alterations in blood flow shear stress, signaling pathways such as NF-κB are strongly activated, driving the high expression of E-selectin on the surface of pulmonary arterial endothelial cells (36). The primary function of E-selectin is to mediate the initial adhesion and rolling of leukocytes (neutrophils, monocytes, and lymphocytes) on the vascular endothelium. In particular, highly expressed E-selectin acts as a “molecular tether” to capture circulating leukocytes and direct their localization to the inflamed vascular endothelium, which is the first step in the extravasation of inflammatory cells into the perivascular tissue (67). The infiltration of these recruited inflammatory cells into perivascular tissues leads to further release of pro-inflammatory and pro-proliferative factors, creating a vicious cycle of endothelial cell activation, E-selectin expression, leukocyte recruitment and infiltration, amplified inflammation, *in situ* thrombosis, and vascular remodeling, which progressively drives disease progression in PAH (74). Accordingly, E-selectin is not only a bridging molecule linking pulmonary artery endothelial cell dysfunction to vascular inflammation in PAH, but also a potential biomarker and a promising target (**Table 1**).

**Table 1:** Summary of the roles of E-selectin in PAH pathogenesis.

Functional mechanism	Cell types involved	E-selectin ligands	Potential signaling pathways	Potential significance in PAH
Leukocyte Recruitment	1.Activated endothelial cells 2.Leukocytes (neutrophils, monocytes, eosinophils,etc.) 3.Smooth muscle cells (interacting with endothelial cells)	PSGL-1 ESL-1 CD44 L-selectin β2-integrin	1.PLCy2/p38 MAPK signaling pathway 2.PI3Ky pathway 3.RAGE-PI3K-AKT pathway	1.Promotes inflammatory cell adhesion and migration into the vascular wall,leading to perivascular inflammation 2.Triggers the release of inflammatory mediators,alters vascular permeability,and promotes thrombosis 3.Induces smooth muscle cell proliferation,migration,and intimal hyperplasia through endothelial cell interactions;contributes to obstructive vascular lesions 4.Enhances prothrombotic state and promotes microthrombus formation in the pulmonary vasculature
Endothelial Cell Signaling				
Vascular Remodeling				
Thrombogenesis				

#### 5.2.2 E-selectin and neutrophils

Recently, the role of the innate immune response, particularly that of neutrophils, in the pathogenesis of PAH has received increasing attention (75). Preclinical studies and patient observations have demonstrated that PAH is associated with a chronic, low-grade inflammatory state, both systemically and locally within the pulmonary vasculature, in which increased numbers and activation of circulating neutrophils, as well as their infiltration into the pulmonary vascular beds, are prominent features (66). Tuder et al. first reported the presence of extensive immune cell infiltration around the pulmonary vasculature in patients with PAH (74). Subsequently, increased numbers of neutrophils were observed in the peripheral blood and lung tissue of patients with PAH and animal models of PAH (76). Following homing to lung tissues during the course of PAH, neutrophils are involved not only in pulmonary vascular inflammation (77), but also in phagocytosis, degranulation, and formation of neutrophil extracellular traps (NETs), and the production of various factors—including neutrophil elastase, myeloperoxidase, cytokines, and reactive oxygen species—that mediate pulmonary vascular remodeling (**Figure 3**) (78, 79). Endothelial cells are activated by inflammatory factors, including IL-1, TNF-α, and LPS. These factors promote NF-κB nuclear translocation via IκB kinase-mediated phosphorylation and degradation of IκB, which in turn upregulates the expression of adhesion molecules such as E-selectin and ICAM-1 (57, 80). In particular, endothelium-derived E-selectin is a critical initiating factor in neutrophil recruitment to the pulmonary vascular wall, mediating leukocyte rolling and initial adhesion by binding to neutrophil surface ligands, including PSGL-1, ESL-1, and β2-integrin (81). This process activates the NF-κB pathway and MAPK signaling pathway in endothelial cells, significantly upregulating the expression of pro-inflammatory factors (IL-6, TNF-α, and IL-1β) and ICAM-1 and VCAM-1 (80, 82). This upregulation further promotes the firm adhesion of neutrophils to the endothelial cell surface and transendothelial migration, which is crucial for driving persistent inflammation and vascular remodeling. Consequently, neutrophils play a pivotal role in the pathophysiological process of PAH (**Figure 3**) (83).

## 6 Exploration of E-selectin-based therapeutic strategies for PAH

Studies have indicated that E-selectin-mediated activation of the NLRP3 inflammasome rapidly stimulate the release of S100A9 from neutrophils (18). S100A9 mediates endothelial cell dysfunction and promotes pulmonary vascular remodeling during PAH through the RAGE-PI3K-AKT signaling pathway (19). Therefore, E-selectin plays a crucial role in the adhesion between neutrophils and endothelial cells, and in the transendothelial migration of neutrophils during the inflammatory response. Targeting the E-selectin/E-selectin ligand axis aims to disrupt neutrophil-endothelial cell adhesion and neutrophil transendothelial migration, thereby inhibiting the inflammatory cascade (84), and ameliorating pulmonary vascular remodeling. However, direct evidence from animal models with E-selectin gene deletion regarding PAH progression or regression remains limited. Existing evidence, primarily derived from inflammation and vascular injury models, suggests that E-selectin deficiency reduces leukocyte rolling, adhesion, and endothelial inflammatory activation (85). Given the upregulation of E-selectin and the pro-inflammatory endothelial phenotype observed in PAH, it is plausible that E-selectin deficiency may delay pulmonary vascular remodeling by attenuating inflammatory cell recruitment and vascular inflammation. Nevertheless, this hypothesis requires direct confirmation using PAH-specific animal models.

### 6.1 Traditional PAH therapeutic drugs

Traditional PAH-targeted drugs act primarily by improving vasodilation, inhibiting smooth muscle proliferation, reducing inflammation, and alleviating endothelial dysfunction. Consequently, they may indirectly affect E-selectin expression or release by lowering inflammation and oxidative stress (86). Current evidence is insufficient to demonstrate that these drugs directly regulate E-selectin; their effects are therefore more likely to reflect a global improvement in endothelial inflammatory status. Despite the progress these drugs have made in relieving symptoms and improving survival, they still have significant limitations. First, they fail to cover all pathological mechanisms and cannot fundamentally reverse vascular remodeling (87). Second, the side effects and safety of these drugs remain significant concerns (88, 89). Third, there are limitations in efficacy and considerable inter-individual variability (89). Finally, the complexity of administration routes and patient compliance pose additional challenges (90). Nevertheless, these drugs remain the cornerstone of current PAH management, and their established clinical efficacy and safety profiles should not be overlooked. With the growing recognition of inflammation in pulmonary vascular dysfunction, immunotherapy has shown great potential in PAH treatment (91). Novel strategies that target immune cells, inflammatory cytokines, and their associated signaling pathways are emerging as promising therapeutic approaches for PAH (92, 93).

### 6.2 Drugs that directly target E-selectin

Existing evidence suggests that anti-E-selectin antibodies can effectively inhibit neutrophil recruitment into injured tissues. In murine models, intravenous administration of anti-E-selectin monoclonal antibodies following intratracheal LPS exposure significantly reduced neutrophil migration into the alveolar space and attenuated acute inflammation (94). Similarly, anti-E-selectin treatment was associated with reduced ischemic injury and neurological dysfunction in a mouse stroke model (95). Nevertheless, E-selectin monoclonal antibodies and related targeting strategies remain largely at the experimental stage, with studies primarily focused on inflammatory vascular diseases, cancer, sickle cell disease, ischemia-reperfusion injury, and transplantation-related inflammation (96–98). To date, no broadly approved clinical indications exist for E-selectin-targeted therapies. In the context of PAH, E-selectin-mediated “active anchoring” systems transcend the limitations of conventional passive targeting by emulating the natural leukocyte adhesion cascade (99). This approach effectively counteracts high mechanical shear stress within pulmonary vessels, ensuring stable drug accumulation at lesion sites. By implementing a hierarchical delivery model—anchoring to the endothelium followed by intercellular transport to smooth muscle cells—this strategy overcomes barriers to drug penetration and enables spatiotemporal control via hypoxia-responsive release. While research remains in its early stages, the ongoing refinement of high-affinity targeting ligands and the rigorous assessment of long-term immunogenicity and safety position E-selectin-guided nanoplatforms as a promising therapeutic paradigm for reversing vascular remodeling and advancing the clinical translation of PAH treatments.

## 7 Discussion and outlook

The strategy of targeting E-selectin holds promise for overcoming the limitations of current PAH therapy. Unlike existing drugs that primarily rely on pulmonary artery vasodilation, this approach is designed to address the core pathological process of vascular remodeling. Although anti-E-selectin therapy can block leukocyte-endothelial adhesion and rolling, thereby suppressing inflammatory cell recruitment and reducing perivascular inflammation and thrombosis, E-selectin also plays a critical role in leukocyte recruitment and host immune defense.

Several key knowledge gaps remain to be addressed. First, the precise temporal and spatial expression patterns of E-selectin during PAH progression have not been fully characterized. Second, whether E-selectin expression levels correlate with disease severity or treatment response in patients with PAH remains unclear. Third, therapeutic efficacy may be constrained by disease stage and by functional redundancy with other adhesion molecules such as P-selectin and VCAM-1. Fourth, the long-term safety profile of E-selectin inhibition, particularly with respect to infection risk, immune function, and potential pro-thrombotic or pro-inflammatory rebound effects upon treatment cessation, remains to be systematically investigated. Therefore, future studies should focus on elucidating the precise role of E-selectin in PAH, optimizing targeting modalities such as antibodies, small molecule inhibitors, nanocarriers, and carefully evaluating off-target effects, immunogenicity, biodistribution, and long-term safety.

## 8 Conclusion

E-selectin, as a core adhesion molecule, initiates inflammatory cascades and is integral to the pathophysiology of PAH. It drives pulmonary vascular remodeling and disease progression through multiple mechanisms, including mediating leukocyte-endothelial cell interactions, amplifying inflammatory responses, inhibiting endothelial cell apoptosis, and promoting phenotypic transformation of pulmonary vascular cells. Evidence from clinical studies indicates that E-selectin expression levels are upregulated in patients with PAH, and its genetic polymorphisms may also be associated with cardiovascular risk. However, several challenges must be acknowledged. The functional redundancy among adhesion molecules may limit the efficacy of E-selectin-specific interventions. The potential risks of long-term immunosuppression and increased susceptibility to infection require careful consideration. Although direct targeting of E-selectin for PAH treatment remains in the exploratory phase, it holds considerable promise as a therapeutic target **—** particularly for inhibiting pulmonary vascular inflammation and thrombosis, and reversing the core pathological process of vascular remodeling. Developing efficient and specific intervention strategies targeting E-selectin, along with an in-depth evaluation of its safety and efficacy, represents a key direction for future research. Overcoming these challenges and gaining a deeper understanding of the complex regulatory network of E-selectin in PAH will provide novel insights and hope for the future treatment of this disease.
